# Sinus mast cell accumulation and persistence suggest a unique endotype in toxin exposure–associated chronic rhinosinusitis

**DOI:** 10.1172/JCI201075

**Published:** 2026-02-10

**Authors:** Xinyu Wang, Yung-An Huang, Anshika Sethi, Christopher Yue, Sydney Brack, Aiswarya Chattuparambil Binoy, Lauren Crowther, Carol Yan, Adam Deconde, Anton Kushnaryov, Andrew Vahabzadeh-Hagh, Jacob Husseman, Maia Little, Allyssa Strohm, Lily Jih, Jonathan J. Lyons, David H. Broide, Taylor A. Doherty

**Affiliations:** ¹ VA San Diego Healthcare System, La Jolla, California, USA.; ² Division of Allergy & Immunology and; 3 Department of Otolaryngology–Head and Neck Surgery, UCSD, La Jolla, California, USA.

**Keywords:** Immunology, Inflammation, Allergy, Mast cells

## Abstract

This study identifies a toxin-associated chronic rhinosinusitis endotype, defined by persistent mast cell accumulation, advancing understanding of how environmental exposures may drive chronic airway disease.

**To the Editor:** Chronic rhinosinusitis (CRS) affects approximately 8%–10% of the general population with an economic burden of billions of dollars (1). Comorbid CRS further significantly increases the severity of asthma (2). Exposure to wildfires and military combustion burn pits leads to inhalation of multiple toxins (3). CRS is increased 3-fold among military veterans with deployment-related exposures to airborne toxins and is associated with more severe symptoms (4). Additionally, nonmilitary CRS patients, including those exposed to combustion exhaust (e.g., mechanics and drivers), require surgery 6 times more frequently for uncontrolled symptoms (5).

CRS is broadly categorized by the presence (CRSwNP) or absence (CRSsNP) of nasal polyps and endotypes based on tissue eosinophils and neutrophils. Currently, there are no FDA-approved therapies for CRSsNP. Mast cells (MCs) are an understudied immune population in CRS and are activated by allergen-IgE binding and non-IgE mechanisms such as cytokines and xenobiotics. The integrin-dependent accumulation and long-lived nature of tissue MCs may promote chronic inflammation (6). Here, we demonstrate enrichment of sinus MCs in burn pit–exposed military veterans with CRS and persistent sinonasal MC accumulation in mice exposed to environmental toxins.

We performed flow cytometry on ethmoid sinus samples obtained from patients undergoing routine sinus surgery (Figure 1, A and B, and Supplemental Figure 1, A and B; supplemental material available online with this article; https://doi.org/10.1172/JCI201075DS1). No difference in tissue eosinophils or neutrophils was observed in deployment exposure CRS samples compared with CRS without exposures. However, there was over a 2-fold increase in sinus MCs (2.829% vs. 1.227%, *P* = 0.0185) in exposed samples primarily from CRSsNP (Figure 1B and Supplemental Figure 1B). Smoking history trended higher in the exposure group (Supplemental Table 1), though multiple regression demonstrated that exposures were an independent predictor of sinus MCs (*P* = 0.0404). There were also more subjects with obstructive sleep apnea in the exposed cohort (69% vs. 10%, *P* = 0.0097). We observed a positive correlation between deployment duration and sinus MC burden (Supplemental Figure 1C). Tissue toluidine blue staining showed increased subepithelial MCs in exposed samples (Figure 1C), and sinus MCs were further increased in exposed patients with positive fungal allergen–specific (*Alternaria*) IgE (Supplemental Figure 1D). Unbiased bulk RNA-seq of exposed sinus tissue revealed upregulation of arachidonic acid metabolism and xenobiotic pathways that are critical to MC function and toxin metabolism, respectively (Figure 1D and Supplemental Figure 1E). Exposed sinuses showed increased expression of the MC-related transcripts *TPSAB1* (encodes tryptase α/β1) and *HDC* (encodes histidine decarboxylase), as well as *ITGB7* (encodes integrin β7), which is critical to MC tissue trafficking (Figure 1D). A mucosal mast cell (MCT) signature suggested MCTs as the prominent MC subset (Supplemental Figure 1F).

To test the effect of combustion-related product compounds (CPCs) on sinonasal inflammation and MC accumulation in vivo, mice received intranasal particulate matter, polycyclic aromatic hydrocarbon, and dioxin, with and without the allergen *Alternaria* (ALT) (Figure 1, E–G). Exposure to ALT or CPCs alone induced submucosal inflammation and epithelial hyperplasia, though these changes were enhanced upon exposure to ALT with CPCs, along with increased subepithelial MCs (Figure 1E). ALT and ALT+CPC-treated mice displayed increases in sinonasal MC numbers at 2 and 4 weeks of exposure, as well as in Integrin B7^+^ MCs, suggesting active recruitment of MC progenitors (Figure 1, F and G). Mouse sinonasal MC identification was confirmed by FACS purification and morphologic assessment (Supplemental Figure 2, A and B). There were no significant differences in tissue neutrophils or eosinophils (Supplemental Figure 2C). In contrast to ALT-challenged mice, total and Integrin B7^+^ MC numbers continued to increase in ALT+CPC-challenged mice for 4 weeks without further challenges (Figure 1F). Male mice also had a more pronounced MC response to ALT+CPC challenge compared with females (Supplemental Figure 2D). This suggests that CPC potentiates allergen-driven sinonasal inflammation and leads to persistent MC accumulation.

Our study demonstrates sinus MC enrichment in veterans with CRSsNP who were previously exposed to airborne toxins during deployment. This finding was independent of smoking history and tissue eosinophils and neutrophils. We also found that coexposure to aeroallergens and toxins in mice led to persistent MC accumulation long after airway exposures. Our work suggests a novel CRS endotype related to previous toxin exposure. Future studies are needed with large CRS cohorts to investigate exposure links to gender, allergic status, and other conditions, including obstructive sleep apnea. These findings have significant clinical implications, including the potential use of MC-targeted therapies in toxin-related CRS.

Detailed methods, biological variables, statistics, study approval, author contributions, acknowledgments, and a list of differentially expressed pathways and genes are provided in the supplemental materials.

## Conflict of interest

The authors have declared that no conflict of interest exists.

## Funding support

This work is the result of NIH funding, in whole or in part, and is subject to the NIH Public Access Policy. Through acceptance of this federal funding, the NIH has been given a right to make the work publicly available in PubMed Central.

- NIH grants (A171795 to TAD; A171795, AI070535, AI242236, and AI107779 to DHB; AI 007469 to XW; and AI138586 to JJL).
- Veterans Affairs (BX005073 to TAD).
- UCSD Stem Cell Program and California Institute for Regenerative Medicine Major Facilities grant (FA1-00607) to the Sanford Consortium for Regenerative Medicine.

## Supplementary Material

Supplemental data

Supplemental data set 1

Supporting data values

## Figures and Tables

**Figure 1:**
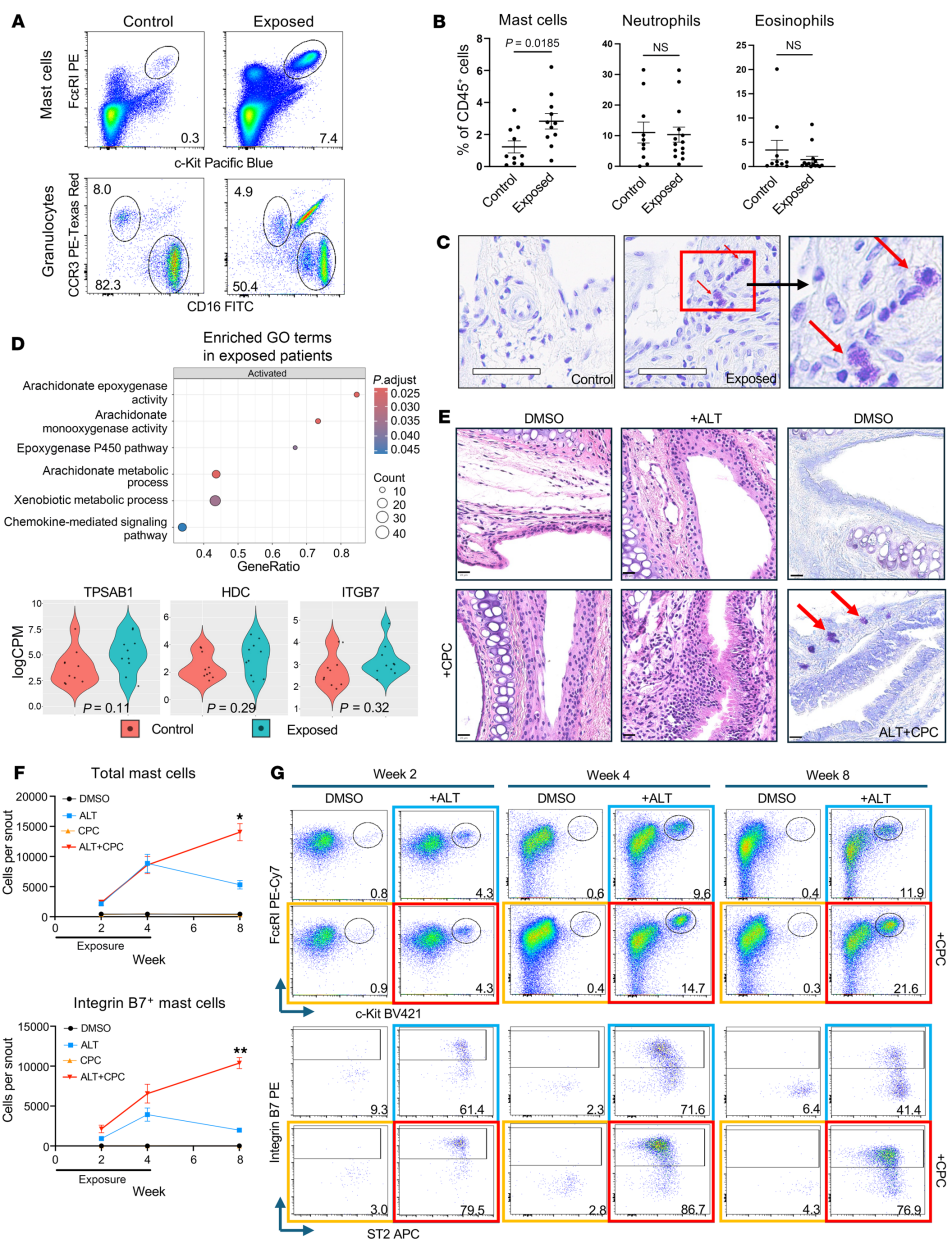
Sinus MCs are enriched in burn pit–exposed military veterans with CRS and in mice exposed to environmental combustion-related compounds. (**A**) Sinus MC gating from control and toxin-exposed tissue. (**B**) Granulocyte percentage of CD45^+^ leukocytes for individual control (*n* = 10) and exposed (*n* = 13) subjects. Data are presented as mean ± SEM; Student’s *t* test. (**C**) Tissue toluidine blue staining. Red arrows indicate MCs. Scale bars = 60 μm; original magnification, ×40. (**D**) Sinus tissue bulk RNA-seq from control (*n* = 10) and exposed (*n* = 10) subjects. GSEA of enriched pathways and violin plots of key MC transcripts with adjusted *P* values shown. (**E**) H&E- or toluidine blue–stained sections of mouse sinonasal respiratory epithelium after 4 weeks of exposure to ALT, CPC, or a combination. Red arrows indicate MCs. Scale bars = 20 μm. (**F**) Total and Integrin B7^+^ MCs in mouse sinonasal tissue over 8 weeks. Data are presented as mean ± SEM for *n* = 4 mice per time point. Mixed-effects model (restricted maximum likelihood) and Tukey’s multiple-comparison test at indicated time points (*P < 0.05, **P < 0.01). (**G**) Representative flow plots of Integrin β7 and ST2 MCs.

## Version 1 02/10/2026

In-Press Preview

## Version 2 04/15/2026

Electronic publication
