# Supplementary material for: Sinus mast cell accumulation and persistence suggest a unique endotype in toxin exposure–associated chronic rhinosinusitis
Source: J Clin Invest. 2026 Feb 10;136(8):e201075. doi: 10.1172/JCI201075 (PMC13078863; doi:10.1172/JCI201075)

Wang et al.

Supplementary Appendix:

Table of Contents

Supplemental methods

Supplementary text

Supplementary table

Supplementary figures

Supplementary data

Supplemental methods

Sex as a biological variable. The human cohort consisted of males (13/13), while the control group included 8 males and 2 females. Due to the nature of the study population, analyses were not stratified by sex. For the preclinical model, female mice were used to increase generalizability. Male mice showed a greater mast cell effect after 2 weeks of toxin and allergen exposure, indicating that female mice provide a conservative estimate of the inflammatory process.

Human Subjects and Sample Collection: Ethmoid sinus tissue samples were obtained from patients undergoing clinically indicated endoscopic sinus surgery for CRS. The study cohort consisted of 23 patients, divided into an "Exposed" group of military Veterans with self-reported deployment-related exposures (n=13) and a "Control" group of Veterans with no known exposures (n=10). Demographic and clinical characteristics were collected from all participants.

Human Tissue Analysis

Flow Cytometry. Fresh sinus tissue was mechanically and enzymatically digested to create a single-cell suspension using Liberase™ (Roche) and DNase. Leukocytes were identified as CD45+. From this population, mast cells were identified as c-Kit (CD117)+ FcεRI+ cells within the CD66b- gate. Eosinophils were identified as CD66b+ CCR3+ CD16- and neutrophils as CD66b+ CCR3- CD16+. A detailed list of antibodies are provided in the supplemental method table.

Histology. Formalin-fixed, paraffin-embedded ethmoid sinus tissue sections were stained with toluidine blue to visualize mast cells.

Bulk RNA Sequencing. RNA was extracted from sinus tissue from a subset of control (n=10) and burn pit-exposed (n=10) patients using RNA miniprep kits (Qiagen). RNA sequencing was performed at the IGM Genomics Center (UC San Diego). RNA sequencing libraries were generated using the Illumina Ribo-Zero Plus rRNA Depletion Kit with Illumina DNA/RNA UD Indexes (Illumina, San Diego, CA). Samples were processed following manufacturer's instructions. Resulting libraries were multiplexed and sequenced with 100 basepair (bp) Paired End (PE100) to a depth of approximately 50 million reads per sample on an Illumina NovaSeq X Plus. Samples were demuxltiplexed using bcl2fastq v2.20 Conversion Software (Illumina, San Diego, CA). Gene Set Enrichment Analysis (GSEA) was performed to identify enriched biological pathways using R studio. RNA was extracted using the RNeasy Mini Kit (Qiagen). RNA sequencing was performed at the IGM Genomics Center (UC San Diego). Reads were aligned to the human genome (GRCh38, GENCODE) with the *Rsubread* package. Differential expression and enrichment analyses were conducted using *edgeR*, *limma*, and *clusterProfiler*.

Animal Studies

Mice. C57BL/6 female mice age 6-8 weeks from Jackson laboratories were used for the following experiments.

Mouse Model of Toxin-Associated Sinonasal Inflammation. Mice (n=4 in each group at each time point) received intranasal challenges three times weekly for four consecutive weeks. Treatment groups included a vehicle control (DMSO), aeroallergen (*Alternaria alternata*, ALT), combustion-related product compounds (CPC), or a combination

(ALT+CPC). The CPC cocktail included 2,3,7,8-Tetrachlorodibenzodioxin, (TCDD; Thermo Fisher Scientific, Waltham, MA), Benzo[a]pyrene (BaP; Millipore Sigma, Burlington, MA), and fine atmospheric particulate matter <4 μm (PM4; National Institute of Standards and Technology, Gaithersburg, MD). The dose of Alt was 30 μg/mouse, and the CPC cocktail was mixed with TCDD (0.6 ng/mouse), BaP (5 ng/mouse), and PM4 (20 μg/mouse). An independent group of mice was monitored for an additional four weeks after the final challenge.

Histological Analysis. Mouse snouts were fixed in 4% PFA, decalcified in 14% EDTA solution for 12 days, and embedded in paraffin. Sinonasal sections were stained with hematoxylin and eosin for morphological assessment or with toluidine blue for mast cell identification.

Flow Cytometry. Total sinonasal tissue was digested to generate single-cell suspensions. Mast cells were identified as CD45+, Lineage-, c-Kit+ FcεRI+ and assessed for co-expression of Integrin β7 and ST2. Eosinophils were identified as CD45+, CD11b+, SiglecF+, Ly6G-. Neutrophils were identified as CD45+, CD11b+, CD11c-, Ly6G+. A subset of SiglecF+ neutrophils were also identified (Supplemental Figure 2A). A detailed list of antibodies are provided in the supplemental method table. Mast cells were sorted using a BD FACSAria Fusion instrument and stained with Wright-Giesma to confirm identification by morphology.

Statistical Analysis. Statistical analyses were performed using GraphPad Prism. For human data, comparisons between two groups were made using unpaired, two-tailed Student's t-test or Mann-Whitney U test for continuous variables and Fisher's exact test for categorical variables. A multiple linear regression model was used to assess the

independent effects of exposure and smoking on mast cell levels. For mouse data, a mixed-effects model (REML) with Tukey's multiple comparisons test was used to analyze differences between groups over time. A P value less than 0.05 was considered significant.

Study Approval. The human study was approved by the Institutional Review Board at VA San Diego Medical Center and at the University of California San Diego. Written informed consent was received from all participants prior to inclusion in the study. All animal experiments were conducted in accordance with protocols approved by the Institutional Animal Care and Use Committee at the University of California San Diego.

Data Availability. The bulk RNA sequencing data reported in this paper have been deposited in Gene Expression Omnibus (GEO) under accession number GSE313186. "Supporting data values" tables have been included in the supplement. All other data are available from the corresponding author upon reasonable request.

Human antibodies			
Fluorophore	Antibody Name	Clone	Manufacturer
FITC	CD16	3G8	Biolegend
PE Texas Red	CCR3	5E8	Biolegend
APC	CD66	QA17A51	Biolegend
APC-Cy7	CD45	2D1	Biolegend
PE	FcEr1	AER-37 (CRA-1)	Biolegend
Pac Blue	C-kit / CD117	W18195C	Biolegend
**Mouse antibodies**			
Fluorophore	Antibody Name	Clone	Manufacturer
FITC	CD11c	N418	BioLegend
PE	Siglec-F	E50-2440	BD Pharmingen
PerCP/Cy5.5	CD45.2	104	BioLegend
APC	CD11b	M1/70	BioLegend
Pacific Blue	Ly-6G	1A8	BioLegend
PE/Cyanine7	Ly-6C	HK1.4	BioLegend
FITC - Lineage	CD4	GK1.5	BioLegend
FITC - Lineage	CD5	53-7.3	BioLegend
FITC - Lineage	CD8a	53-6.7	eBioscience
FITC - Lineage	CD19	6D5	BioLegend
FITC - Lineage	CD11c	N418	BioLegend
FITC - Lineage	NK-1.1	PK136	BioLegend
FITC - Lineage	CD3/Gr-1/CD11b/CD45R(B220)/Ter-119	[n/a]	BioLegend
Brilliant Violet 421	C-kit / CD117	2B8	BioLegend
PE/Cyanine7	FcεRIα	45717	BioLegend
Brilliant Violet 650	CD45.2	104	BioLegend
PE	Integrin β7	M293	BD Pharmingen
APC	IL-33Rα (IL1RL1, ST2)	DIH9	BioLegend
APC/Cyanine7	CD69	H1.2F3	BioLegend

Author Contributions. XW designed research studies, conducted experiments, acquired data, analyzed data, and wrote the manuscript. A. Sethi, A. Strohm, AC, SB, CY, YH, and LC conducted experiments and acquired data. CY, AD, AK, AVH, and JH provided human samples. ML, A. Sethi, and LJ analyzed data. DB and JJL assisted with manuscript writing and experimental design. TD designed research studies, analyzed data, and wrote the manuscript.

Acknowledgments.

This publication includes data generated at the UC San Diego IGM Genomics Center utilizing an Illumina NovaSeq X Plus that was purchased with funding from a National Institutes of Health SIG grant (#S10 OD026929). We are grateful to Jennifer Santini and the UCSD SOM Microscopy Core (NS047101, OD030505, OD036455). We also thank Cody Fine and Mitra Banihassan (UCSD) for technical assistance with flow cytometry experiments. This work was additionally made possible by the UC San Diego Stem Cell Program and a CIRM Major Facilities grant (FA1-00607) to the Sanford Consortium for Regenerative Medicine. This publication includes data generated at the UCSD Human Embryonic Stem Cell Core Facility, using the FACS Aria Fusion and FACS Aria II Flow Cytometry Sorters.

Supplementary legends

Supplementary Table 1. Demographic information collected from Veterans with CRS who had self-reported burn pit exposures and Veterans who reported no known exposures. Data are presented as n (%) for categorical variables or median with interquartile range for continuous variables. Statistical comparisons between the Exposed and Control groups were performed using Fisher's exact test for categorical data and the Mann-Whitney U test for continuous data.

Supplementary Data Set 1. List of all enriched biological pathways on GSEA analysis and differentially expressed (DE) genes between Exposed and Control patient samples.

Supplementary Figure 1.

(A) Representative gating strategy for identifying human sinus mast cells, neutrophils, and eosinophils. Single cells were selected based on forward and side scatter (FSC-A, SSC-A), and leukocytes were identified as CD45+. From the CD45+ population, granulocytes were identified as eosinophils (CD66b+ CCR3+ CD16-) and neutrophils (CD66b+ CCR3- CD16+). Mast cells were identified from the CD45+ CD66b- gate as cells positive for both c-Kit (CD117) and FcεRI and confirmed with cell sorting followed by Wright-Giesma stain. Numbers indicate the percentage of cells relative to the parent gate.

(B) Quantification of mast cells, shown as a percentage of total CD45+ leukocytes, in Control (n=10) versus Exposed (n=11) subjects. Data are stratified by chronic

rhinosinusitis (CRS) subtype: CRS without nasal polyps (CRSsNP, left) and CRS with nasal polyps (CRSwNP, right). Each dot represents an individual subject, with lines indicating the mean and standard error of the mean (SEM). P-values are indicated above each comparison.

(C) Correlation analysis of mast cell percentage from exposed individuals with the duration of deployments (in months). The solid line represents the best fit from a simple linear regression. The coefficient of determination (R2) and p-value for each correlation are shown.

(D) Comparison of mast cell percent in exposed individuals with positive Alternaria IgE.

(E) Heatmap of xenobiotic pathway and arachidonate metabolic pathway genes

(F) Heatmap of MCT (mucosal) and MCTC (connective tissue) mast cell genes with MCT predominance index calculated as log ratio of TPSAB1 over CMA1 counts per million.

Supplementary Figure 2.

(A) Representative flow cytometry gating strategy for identifying murine mast cells, neutrophils, eosinophils, and monocytes from total sinus tissue. The gating strategy for mast cells (top row) and other myeloid cells (bottom row) is shown. Numbers adjacent to gates indicate the percentage of cells within the parent gate.

(B) FACS purified sinonasal mast cells from mice challenged with intranasal ALT+CPC followed by Wright-Giesma staining.

(C) Quantification of eosinophils (top) and neutrophils (bottom) per sinus over an 8-week period. Mice were exposed for 4 weeks, followed by 4 weeks of rest. Data are presented as mean ± SEM for n=4 mice per group. Statistical analysis was performed using a mixed-effects model (REML), followed by Tukey's multiple comparisons test.

(D) Comparison of sinonasal mast cell numbers in male vs female mice challenged with intranasal ALT+CPC for 2 consecutive weeks. Student's t-test.

Supplementary Table 1. Patient Characteristics

Patients (n = 23)	Exposed (13)	Control (10)	p-value
**Median age** (years)	47 (40 – 55)	52 (39 – 65)	0.4736
**Sex**			
Male	13 (100%)	8 (80%)	0.1779
Female	0	2 (20%)	
**Race**			
White	10 (77%)	8 (80%)	
Black	0	0	
Asian	3 (23%)	2 (20%)	>0.9999
American Indian/Alaskan Native	0	0	
Declined to answer	0	0	
**Ethnicity**			
Hispanic	4 (31%)	1 (10%)	
Non-Hispanic	9 (69%)	8 (80%)	0.3394
Declined to answer	0	1 (10%)	
**Smoking history**			
Never	3 (23%)	6 (60%)	0.1023
Previous/current use	10 (77%)	4 (40%)	
**Median packyears**	1.4 (0.05 – 12.25)	0 (0 – 2.0)	0.067
**Comorbidities**			
Asthma or COPD	1 (7%)	2 (20%)	0.5569
Allergic rhinitis	7 (54%)	3 (30%)	0.4015
OSA	9 (69%)	1 (10%)	0.0097
GERD	4 (31%)	1 (10%)	0.3394
**CRS with nasal polyposis**	4 (31%)	3 (30%)	>0.9999
**Lund-Mackay (CT) Score**	9 (2.75 – 12.0)	12 (8.5 – 15)	0.1820
**SNOT-22 questionnaire** (# answered)	6	8	
Median score	69 (57-78)	33 (20 - 52)	0.0270
**Medical Therapy**			
Intranasal corticosteroids	11 (79%)	9 (90%)	
Antihistamines (oral/topical)	12 (86%)	5 (50%)	0.5138
Corticosteroid sinus irrigation	3 (21%)	4 (40%)	
Biologics (Dupilumab, etc.)	1 (7%)	0	
**Inhalation exposures**			
Burn Pits	13 (100%)		
+ Diesel/Jet fuel	10 (71%)	n/a	
+ Fires	4 (31%)		
+ Sandstorms	8 (62%)		
**Exposure duration (months)**	4.0 (1.0 – 9.0)	n/a	
**Time since last exposure (years)**	19 (10.5 – 31.5)	n/a	
**Deployment History**			
Middle East	7 (50%)	0	
Europe	3 (21%)	0	
Asia	6 (43%)	0	
Africa	2 (14%)	0	
South America	1 (7%)	0	
North America	1 (7%)	0	
Never deployed	0	10 (100%)	
**Deployment duration (months)**	9.0 (1.75 – 12.75)	0	

Supplementary Figure 1

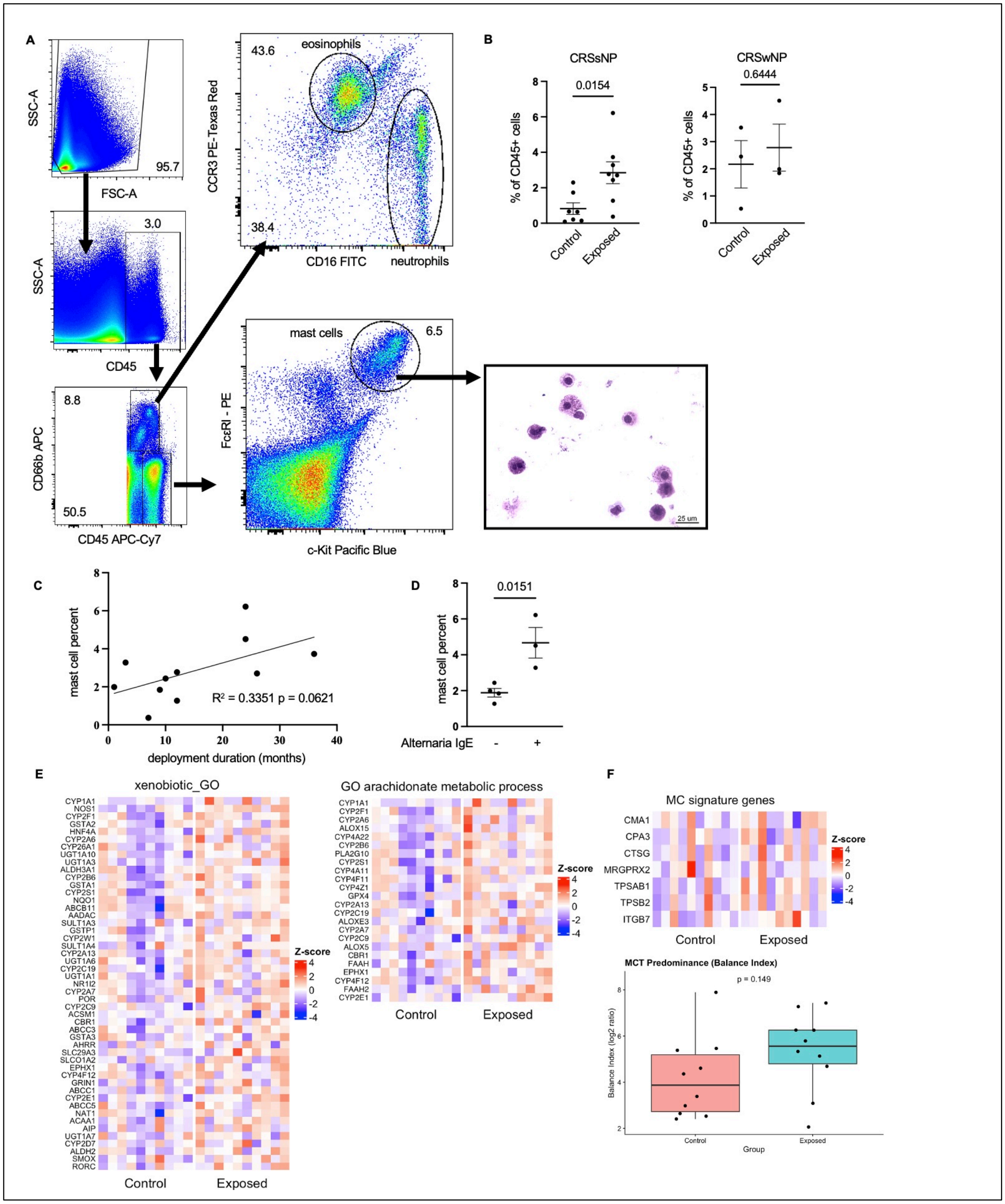

Supplementary Figure 2

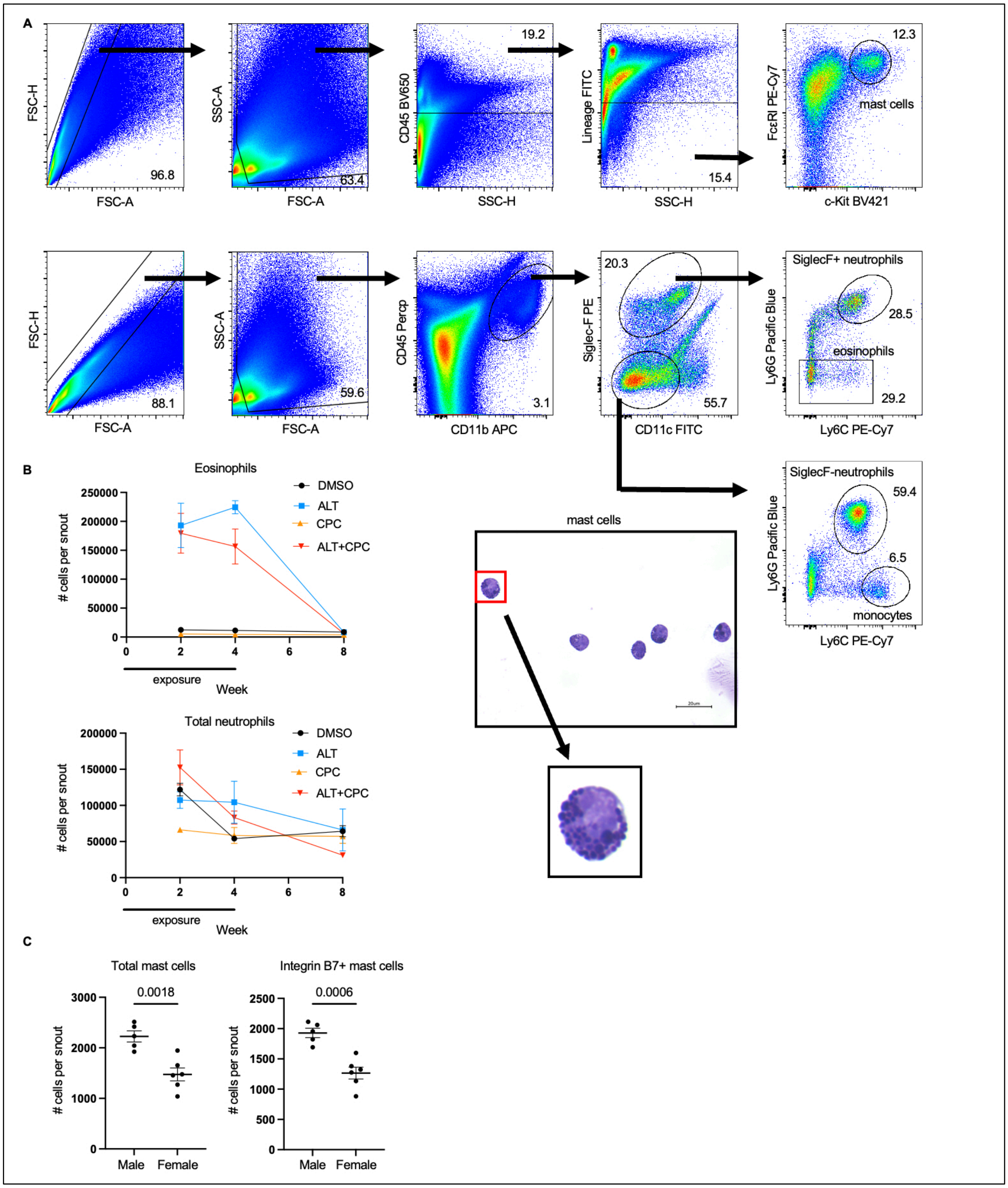

Supplement: Supplemental data [file jci-136-201075-s158.pdf]
